# Advancing Understanding of Dermatological Manifestations in Munchausen Syndrome by Proxy

**DOI:** 10.7759/cureus.71616

**Published:** 2024-10-16

**Authors:** Kelly M Frasier, Haily A Fritts, Vivian Li, Christina Dudzik, Michelle Sobotka, Kathleen Click, Alexandra Loperfito

**Affiliations:** 1 Dermatology, Northwell Health, New Hyde Park, USA; 2 Dermatology, Idaho College of Osteopathic Medicine, Meridian, USA; 3 Medical School, Lake Erie College of Osteopathic Medicine, Erie, USA; 4 Dermatology, Touro College of Osteopathic Medicine, Middletown, USA; 5 Dermatology, Midwestern University Arizona College of Osteopathic Medicine, Glendale, USA; 6 Dermatology, Kansas City University of Medicine and Biosciences, Kansas City, USA; 7 Dermatology, Edward Via College of Osteopathic Medicine, Blacksburg, USA

**Keywords:** child abuse, child abuse and munchausen by proxy, factitious disorder by proxy, factitious disorder imposed on another, munchausen's disorder, munchausen syndrome by proxy, munchhausen by proxy

## Abstract

This comprehensive review critically examines the complex relationship between Munchausen Syndrome by Proxy (MSBP) and its dermatological manifestations, uncovering new insights into the relatively uncharted territory of this psychiatric disorder. By synthesizing existing literature, case studies, and clinical observations, this paper reveals the multifaceted spectrum of dermatological phenomena seen in individuals affected by MSBP, ranging from self-inflicted injuries to entirely fabricated skin conditions. Additionally, we explore the psychological and behavioral mechanisms driving caregivers to induce or stimulate dermatological symptoms, shedding light on underlying psychopathologies, the nuanced dynamics of the caregiver-patient relationship, and patterns of pathological attachment and dependency. This review confronts the significant obstacles healthcare providers encounter in accurately identifying dermatological symptoms related to MSBP amidst genuine dermatologic conditions, underscoring the indispensable role of a multidisciplinary strategy and heightened clinical vigilance in these complex cases. Future investigations call for the development of standardized assessment tools, the potential of biomarkers for early detection, the exploration of the neurobiological basis of MSBP, and the utilization of specific interventions to protect susceptible populations while improving the accuracy of MSBP diagnoses. This paper calls for a more informed, sensitive, and proactive approach to better understanding and treating the dermatological manifestations of MSBP.

## Introduction and background

Munchausen Syndrome by Proxy (MSBP), recently renamed Factitious Disorder Imposed by Another, is a highly complex psychiatric condition that presents numerous challenges in diagnosis and treatment. The Diagnostic and Statistical Manual of Mental Disorders (DSM)-V defines MSBP as the falsification and fabrication of disease imposed on another individual without evidence of external incentives, which distinguishes MSBP from malingering by proxy [1]. MSBP typically presents as a guardian or caregiver presenting their child or dependent as ill, impaired, or injured. These individuals commonly display fraudulent behavior to support their claims, such as providing falsified lab results [1,2]. These behaviors occur in single or repeated episodes, in which nonspecific pain and symptoms prevail. MSBP most commonly occurs as parent-to-child infliction of falsified disease and accounts for approximately 0.04% of child abuse cases [2]. Abdurrachid et al. reveal that almost all perpetrators of MSBP are female, and nearly 30% have been previously diagnosed with a concurrent psychiatric condition [3]. Early recognition and intervention are critical to preventing the perpetrator from inflicting further abuse on the victim. A multidisciplinary approach involving healthcare providers, social services, and law enforcement is essential for both diagnosing the disorder and ensuring the safety of the affected individuals.

MSBP commonly presents with the perpetrator inflicting a variety of fabricated symptoms on their victim, the most common fabricated symptoms being abdominal pain, recurrent pain in multiple sites, unexplainable metabolic disorders, and unexplained bleeding [1,4]. The perpetrator of MSBP not only fabricates symptoms in their victim, but the psychological abuse can be so severe that the victim may begin to experience real distress or physical symptoms. In certain cases, MSBP can present cutaneously, which poses unique challenges in an already complex psychological disorder. Dermatologic manifestations of MSBP, such as non-healing wounds and rashes, require specialized care and attention from healthcare providers, including dermatologists. Dermatologists are skilled in identifying and treating skin conditions and diseases. However, given the rarity of MSBP and the limited psychiatric exposure in dermatological training, it can be challenging for them to recognize the signs and symptoms of MSBP. This can result in missed detection and misdiagnosis, leading to ongoing abuse and false disease attribution to the victim. This review offers a thorough overview of dermatological manifestations observed in MSBP. By utilizing existing literature, such as case studies and clinical observations, we provide a resource to assist dermatologists in thoroughly evaluating and diagnosing cutaneous MSBP in cases with a high clinical suspicion of fabricated components to a patient’s presentation.

## Review

Psychosocial aspects of MSBP

MSBP can manifest in various ways within the healthcare setting, making it crucial for physicians to promptly identify and rule out life-threatening conditions while also investigating the psychosocial factors contributing to the patient’s symptoms. The victim is often impacted both psychologically and medically due to unnecessary medical testing and treatments they are often subjected to, as well as potential domestic abuse. If the victim is a child, MSBP often interferes with their education, development, and daily life [5,6]. The caregiver imposing a sick role on their child commonly results in frequent school absences and, in extreme cases, complete withdrawal from school. These frequent absences and inflicted medical symptoms can also lead to social isolation from peers, confusion about their condition, and anxiety about their health. Due to their innate trusting relationship with their caregiver, the child often embraces their belief and fully embodies their fabricated illness [7]. The prolonged exposure to these fabricated illnesses can cause the child to internalize the false narrative, leading to a distorted self-perception and a reduced ability to differentiate between genuine and imposed symptoms. The sick role that the child victim assumes also results in increased dependency on the caregiver. Over time, this can severely impact the child's emotional and psychological well-being, increasing the risk of long-term mental health issues such as anxiety, depression, and traumatic stress.

Understanding the caregiver's motivation and psychological dynamics is essential in recognizing how medical professionals should approach MSBP cases. Glaser denotes that the caregiver’s motivation often originates from a desire to receive increased sympathy and validation from a medical professional by confirming the caregiver’s concerns for the patient [5,6]. It is likely to see accompanying mental health disorders in caregivers; specifically, generalized anxiety disorder may contribute to unfounded concerns regarding the patient. In addition, personality disorders such as borderline, histrionic, or mixed type may contribute to the deceptive component of MSBP [3]. The dynamics of MSBP typically involve a triad of individuals: the caregiver, the patient, and the medical professional. The caregiver fabricates the medical situation, leading the patient (often a child) to be deceived into believing that they are suffering from a serious health issue that requires medical treatment. This manipulation fosters a false sense of illness in the victim, causing them to seek unnecessary medical attention and further reinforcing the caregiver's control over their well-being. Medical professionals, though unknowingly, can become complicit by conducting unnecessary tests or treatments, inadvertently perpetuating the disorder. The role of medical professionals in this dynamic often revolves around satisfying the caregiver's need for validation by conducting extensive investigations and medical tests. Unfortunately, these actions can unintentionally harm the patient through unnecessary and invasive procedures and increase the caregiver’s desire to continue the cycle [8]. Due to this risk, medical professionals need to remain vigilant for signs of MSBP and recognize the potential harm it can inflict on the patient.

In addressing MSBP, early intervention, and a multidisciplinary approach are critical for breaking the cycle of abuse and safeguarding the victim's well-being. Collaboration among healthcare providers, social workers, and mental health professionals is essential to accurately diagnose the condition and prevent further harm. Once MSBP is suspected, healthcare professionals must approach the case with a high degree of sensitivity, balancing the need to protect the victim with the legal and ethical complexities involved in confronting the caregiver. This may involve careful documentation, covert surveillance, or even covert hospital admissions to observe discrepancies between reported symptoms and the child’s actual condition. The involvement of child protective services and law enforcement is often necessary, as legal action may be required to remove the victim from the harmful environment. Ensuring that the child receives comprehensive psychological care is vital in helping them recover from the trauma, rebuild their self-perception, and learn to differentiate between fabricated and genuine health concerns. Furthermore, educating healthcare providers about the signs of MSBP and establishing clear protocols for identifying and managing suspected cases can prevent further abuse and protect future victims from the physical and psychological ramifications of this insidious condition.

Dermatological manifestations of MSBP

Self-inflicted or caregiver-inflicted skin conditions can present in a variety of ways, often complicating diagnosis due to their unusual or inconsistent patterns. Injuries can range from excoriations, ulcers, and burns to deeper wounds that appear resistant to healing. Three specific terms describe the dermatological manifestations of Munchausen syndrome and Munchausen syndrome by Proxy, with the distinction in the intent behind the behavior. The lesions in each of these conditions are often present in unusual shapes, such as linear or geometric patterns, or involve skin damage that does not align with typical dermatological diseases. While the visual presentation may be consistent across these terms, they differ in the underlying psychological drivers behind the behavior. Dermatitis factitia, or factitious dermatitis, involves the deliberate infliction of injury to the skin by the patient, which is primarily driven by psychological distress or psychiatric conditions [9]. The patient is aware of their actions but may not fully understand why they are doing it. Dermatitis artefacta is closely related to and often used interchangeably with dermatitis factitia; however, the patient is fully aware of their actions, and the intent is often to deceive medical professionals, potentially to gain attention or sympathy [10]. In the same way, these lesions are unusual in shape and distribution and do not align with common dermatological diseases. Finally, dermatitis simulata refers to skin lesions intentionally created or altered by a patient or caregiver to mimic a legitimate dermatological condition [11]. This is often done by the patient or caregiver to deceive healthcare professionals. This condition is more aligned with intentional fabrication, where the goal is often to convince others of a medical condition that doesn’t exist, sometimes for secondary gains such as attention or other psychological needs. 

Chiriac et al. describe two patients suffering from factitious dermatitis with the first case involving a 77-year-old woman with undiagnosed psychiatric issues who had self-inflicted atrophic skin lesions on her face for several months [12]. Despite multiple hospital admissions in dermatology units and various treatment approaches, there was no improvement until she received psychiatric treatment for major depressive syndrome, leading to full recovery. The second case was a 61-year-old woman with widespread atrophic scars on her face, trunk, and limbs. Her history of a suicide attempt raised concerns about underlying psychiatric conditions, and after being prescribed antidepressants, she showed significant clinical improvement in her dermatological symptoms. In both of these patient cases, there was no malicious intent of deception but a need to fulfill psychological urges. Hariharasubramony et al. describe a case of dermatitis simulata secondary to Munchausen syndrome in a twenty-eight-year-old woman with wounds presenting as red-brown macules of similar size and morphology throughout the body, most prominently seen on the lower extremities [11]. This patient had a history of multiple hospital admissions due to unexplained illness and, each time, left in the middle of treatment. After undergoing psychiatric evaluation and closer inspection of the wounds, the patient was diagnosed with dermatitis simulata. The frequent hospital visits, persistent nonhealing wounds, and failure to adhere to treatment suggested a more deliberate intent on the part of the patient.

Multiple reports have shown that MSBP dermatologic findings may present similarly to granuloma annulare, cicatricial pemphigoid, recurrent nail avulsion, purpura, and coagulopathy. Each condition can be challenging for dermatologists to diagnose, as the lesions may resemble common skin diseases such as infections, autoimmune disorders, or allergic reactions. However, inconsistent clinical histories, unusual distribution of lesions, and a lack of response to conventional treatments often raise suspicion of these factitious disorders. Sirka et al. reported a fifteen-month-old girl presented with multiple dermatologic findings, including recurrent blisters rupturing to form linear ulcerations on her scalp and cheek, diffuse bizarre-shaped hyperpigmented and hypopigmented lesions, patchy cicatricial alopecia on her scalp, and ectropion of her left upper eyelid [13]. The child had previously been diagnosed with epidermolysis bullosa; however, on further evaluation, these findings were found to be more consistent with inflicted injuries. On further questioning, the mother was the parent to always notice these injuries, and they only occurred when the father was absent. The wounds began to smell of different oils as the injuries progressed. The child was separated from their mother, and when hospitalized, no new injuries appeared. Both parents received psychiatric counseling to address the psychological factors influencing the mother's actions. This case highlights the importance of thorough questioning regarding the patient’s presentation to arrive at the correct diagnosis and protect the victim from further harm inflicted by the caregiver. 

Dermatologists play a crucial role in recognizing the dermatological manifestations of MSBP, as these skin-related symptoms are often pivotal in diagnosing this complex condition. Their ability to identify patterns of self-inflicted or caregiver-induced skin lesions, particularly those that do not align with typical dermatological conditions, can provide essential clues to uncovering the underlying psychiatric disorder. Unusual lesion patterns, non-healing wounds, repetitive injuries, or lesions in areas that are difficult for the patient to reach, may signal the need for a deeper investigation. By recognizing these warning signs and inconsistencies in the clinical presentation, dermatologists can play an instrumental role in initiating further evaluations that lead to the identification of MSBP and the necessary multidisciplinary interventions to protect the patient from ongoing harm. 

In addition to recognizing the clinical manifestations, dermatologists must also remain vigilant for certain behavioral red flags that may accompany MSBP cases. The caregiver often appears overly involved in the medical process, providing detailed and sometimes exaggerated descriptions of the patient's symptoms while showing little concern for the emotional distress or invasive treatments the child endures. This heightened medical engagement is sometimes paired with frequent requests for unnecessary diagnostic procedures or referrals to multiple specialists, despite a lack of conclusive findings. In many instances, the caregiver may seem overly eager for the child to undergo invasive tests or surgical interventions, reinforcing their role as the primary authority on the patient's condition. This behavior can further complicate diagnosis and treatment, as healthcare providers may feel pressured to comply with the caregiver's demands out of fear of missing a serious condition. Moreover, when healthcare professionals question the caregiver’s narrative or suggest a psychiatric evaluation, they may encounter resistance or hostility, with the caregiver seeking alternative opinions or transferring the patient to another facility. This reluctance to follow through with suggested treatments or diagnostics aimed at psychological or psychiatric evaluation is a critical indicator of MSBP and should prompt further scrutiny. Recognizing these behavioral patterns, in conjunction with the dermatological findings, is key to unveiling the true nature of the condition and ensuring that appropriate medical, legal, and protective measures are taken.

Approach to identifying and managing MSBP

Documented cases of MSBP have provided valuable insight into our current understanding and management of MSBP with dermatological manifestations. A case report by Nico and Dwan describes a 45-year-old man who presented with intense dermatological symptoms of stinging, burning, and pain that he claimed were from a worm infection in his skin [14]. Upon physical examination, the lesions consisted of bizarre-shaped lesions and scars created by his wife manipulating the skin to look for these worms. The dermatologic team initially diagnosed the patient with Folie a deux. However, treatment with risperidone and, subsequently, olanzapine did not alleviate the symptoms. During separate psychiatric interviews with the patient and his wife, the patient claimed to be in intense distress from these symptoms, yet he showed no signs of distress and maintained a calm demeanor throughout the entire interview. His wife, however, remained adamant that her husband continued to suffer from a parasitic infection [14]. The dynamics of this situation led the dermatology team to diagnose the patient with MSBP, with the wife as the perpetrator. This case demonstrates the complexity of MSBP, with the psychiatric state of the perpetrator being at the center of the syndrome and the victim's susceptibility to persuasion being a vital factor in the manifestation of MSBP. 

Dermatologists often struggle with identifying MSBP, as the dermatological manifestations vary widely, and the diagnosis is only considered if there is high clinical suspicion. Many dermatologic differentials focus on cutaneous presentations, whereas the physician aims to rule in or out erythematous, ulcerative, or gangrenous pathologies. Once the patient is medically cleared, MSBP becomes a diagnosis of exclusion, especially when the patient’s history does not seem cohesive with their presentation. Suspicion of deception is required, along with contextual cues from fabricated stories obtained from multiple sources [15]. It can be challenging to directly observe the perpetrator in the act of abusing the victim. Therefore, an extensive review of medical records is needed to diagnose MSBP. MSBP may be suspected as the primary diagnosis based on the patient’s presentation and behaviors. Each case of MSBP requires unique and multifaceted treatment that prioritizes patient safety, treating remaining medical concerns, and providing psychiatric help to the patient, perpetrator, and their other family member. Early identification of MSBP is crucial for improving patient outcomes and minimizing the unnecessary use of time and healthcare resources [16]. Establishing a good relationship with patients and their families can help decrease becoming lost to follow-up and ensure treatment compliance.

The use of a multidisciplinary team in the diagnosis of MSBP is crucial, especially in cases of child involvement. When a diagnosis of MSBP is made based on circumstantial evidence, the perpetrator is often not confronted regarding their falsifications directly. Often, a medical professional will approach the caregiver, informing them there is no medical basis for the victim’s symptoms. It is recommended that other caregivers for the victim are also present for the delivery of this information if possible [17]. With cases of a child victim, it is imperative to involve Child Protective Services and a legal team in the management and intervention of MSBP [18]. The most crucial element in MSBP is intervening for the victim’s safety and facilitating treatment for both the perpetrator and the victim. Per Roesler and Jenny, the actions physicians can take to help identify MSBP and establish a multidisciplinary team include carefully documenting patient and caregiver history, comparing histories, exam findings, and original lab documentation with all treatment team providers [19]. Compilation of records enables healthcare providers to analyze the perpetrator’s pattern of actions and the victim’s prior symptoms. Following the diagnosis of MSBP, a psychological review of the caregiver is necessary to determine if any comorbid psychiatric illnesses are present [17]. Mental health professionals screen both parties for comorbid psychiatric conditions and support them with effective treatment modalities. Reunifying the victim with the caregiver is possible if the caregiver can acknowledge their actions, work closely with health and social services, and is not at risk for compromising the victim’s safety [15]. The ultimate goal in the entire process remains the safety of the victim. 

Diagnosing and managing MSBP involves navigating complex legal and ethical dilemmas, particularly regarding the caregiver’s rights and the victim’s welfare. The ethical challenge lies in balancing the need to protect the child while respecting the caregiver’s autonomy and privacy. Confidentiality concerns, the potential for false accusations, and the impact of intervention on the caregiver-child relationship must be carefully considered. Ethical frameworks should guide healthcare professionals to prioritize the victim’s safety while respecting the caregiver’s rights. Ongoing dialogue and ethical training are essential in the context of MSBP. The legal aspects of MSBP involve mandatory reporting requirements, the protection of children, and the prosecution of offenders. Jurisdictions vary in their legal definitions and responses to MSBP, influencing the protocols for reporting suspected cases. Developing clear and consistent legal guidelines is necessary to ensure that healthcare professionals can act in the child’s best interest without fear of legal retaliation for reporting. Research into the effectiveness of current legal frameworks and the experiences of professionals in reporting MSBP can inform policy revisions to better protect children.

In addition to the ethical and legal challenges, diagnosing MSBP requires healthcare professionals to remain vigilant for subtle cues that extend beyond the medical history and physical examination. Psychological manipulation is a key component of MSBP, and perpetrators often exhibit sophisticated tactics to control the narrative. They may present themselves as model caregivers, meticulously involved in the victim’s care, while simultaneously fabricating or exaggerating symptoms to elicit medical intervention. This deception can make it difficult for clinicians to identify the underlying psychological motive, particularly when the perpetrator appears cooperative and compassionate. However, inconsistencies in the patient’s medical history, exaggerated symptomatology that doesn’t align with clinical findings, and frequent hospital visits for unexplained symptoms are red flags that should prompt a deeper investigation. Further complicating diagnosis is the fact that perpetrators often possess extensive medical knowledge, which enables them to fabricate symptoms in a way that mimics real conditions, thus delaying accurate diagnosis and treatment. Advanced strategies, such as covert video surveillance or the use of hidden cameras in hospital settings, have sometimes been employed in extreme cases to catch perpetrators in the act of inducing harm. Although ethically controversial, these techniques can provide critical evidence for establishing MSBP when other methods fail. Given the complexity and potential for harm in these cases, early collaboration with psychiatric and social services is essential to ensure that both the patient and perpetrator receive appropriate interventions while maintaining the integrity of the diagnostic process.

Future directions in research

Creating standardized assessment tools is pivotal for advancing the diagnosis and management of MSBP. These tools would allow for more objective, reliable criteria to identify MSBP by addressing challenges distinguishing between genuine medical conditions and those fabricated or induced by caregivers. Developing such instruments requires multidisciplinary collaboration to integrate medical, psychological, and social parameters, ensuring a comprehensive caregiver and child assessment. Future research must focus on validating these tools across diverse populations and settings to ensure their broad applicability and sensitivity in detecting subtle signs of MSBP. By standardizing the assessment process, healthcare providers can more readily identify suspected cases, leading to timely and appropriate interventions. 

More studies are needed to examine the neurobiological development of perpetrators closely. There is a lack of data on caregivers as most reports focus on the abuse suffered by the victim. A review by Yates and Bass, based on the characteristics of 796 perpetrators, determined that perpetrators are usually young adults, with a mean age of 26.7 at the child presentation, predominantly female (97.6%), married (75.8%), and the mother of the victim (95.58%) [20]. Perpetrators often have accompanying psychiatric conditions, such as personality disorders (18.6%), depression (14.2%), and factitious disorders imposed on self (30.9%). A significant number of perpetrators also endured psychosocial challenges, including childhood maltreatment (30%) and obstetric complications (23.5%) [20]. These characteristics indicate that there is potentially a neurobiological reason for the development of MSBP. Early childhood psychological stress and trauma have been shown to significantly impact brain structural development and, ultimately, function [21]. In cases of MSBP, perpetrators often place their victims in compromising situations to satisfy their own need for sympathy and attention, potentially stemming from their prior abuse [20]. The significant neurodevelopmental impact of childhood trauma may predispose individuals to becoming perpetrators of MSBP.

Identifying biomarkers for early detection of MSBP presents a promising avenue for preventing long-term harm to victims. Research should investigate physiological or biochemical markers indicating stress responses, inflammation, or other signs consistent with fabricated or induced illnesses. For example, elevated stress hormones or unique immune markers in children could signal underlying psychological or physical abuse [22]. Prospective studies are necessary to explore the relationship between potential biomarkers and MSBP, aiming to establish a reliable, non-invasive method for early identification of at-risk individuals. While functional neuroimaging has been explored based on the neurobiological impact implicated in MSBP, few cases are available that aim at detecting deceit [23]. Neuroimaging aims to properly diagnose factitious pathology by monitoring brain activity for signs of deceit while answering questions. When individuals lie, brain activity increases in the prefrontal and anterior cingulate cortex [18, 23]. However, there are many ethical and legal issues regarding the widespread use of neuroimaging to diagnose MSBP. Advancements in this field could be used in combination with other psychiatric diagnostic tools to identify a patient with MSBP. These resources would be especially helpful in making diagnoses in cases without clear evidence of the perpetrator’s abuse.

The implementation of targeted interventions and preventive measures is crucial for protecting children from the harms that arise through being a victim of MSBP. Interventions should focus on education, support, and therapy for caregivers, especially those identified as at risk due to mental health issues or stressful life circumstances. These therapeutic approaches should aim to address the underlying psychological issues in caregivers, such as attachment disorders, personality disorders, and a history of abuse or neglect. Preventive measures could include training healthcare professionals to recognize early signs of MSBP and establishing protocols for multidisciplinary interventions. Research should evaluate the effectiveness of these strategies in real-world settings, adapting interventions based on feedback and outcomes to optimize child welfare. Additional research should also explore genetic predispositions to mental health disorders, personality disorders, or stress responses contributing to the development of MSBP. 

The goal of both research and practice in the context of MSBP is the protection of vulnerable populations. This involves not only direct interventions to safeguard children but also broader societal efforts to address the root causes of the disorder, such as caregiver mental health issues, social isolation, and lack of access to support services. Strengthening social safety nets, providing mental health resources, and fostering community awareness of MSBP are vital strategies. Research investigating effective methods for protecting vulnerable populations will support the development of comprehensive, multidisciplinary approaches to prevent MSBP and facilitate the protection of children’s well-being.

Exploring the role of healthcare professionals in the identification and management of MSBP is essential to preventing ongoing abuse and ensuring victim safety. Physicians, nurses, social workers, and mental health professionals must work together to develop heightened awareness of MSBP’s warning signs and improve clinical training on this rare but damaging form of abuse. Frontline medical staff are often the first to encounter patients presenting with fabricated or exaggerated symptoms, making their ability to detect discrepancies in medical histories and clinical findings critical. Training programs should emphasize not only the medical indicators of MSBP but also the psychosocial dynamics that underpin the syndrome, such as the caregiver’s motivation and behavioral patterns. Additionally, implementing systematic training modules on MSBP detection within medical school curricula and continuing education for licensed professionals can enhance diagnostic accuracy. Medical professionals should also be encouraged to document suspicions carefully and communicate with interdisciplinary teams early in the diagnostic process. Enhanced training could help avoid potential misdiagnoses, such as confusing MSBP with other psychiatric or dermatological conditions, and foster an environment in which early intervention is possible. Given the complexity of MSBP, creating robust, standardized educational resources across healthcare professions could substantially improve the ability to detect and manage cases before significant harm is inflicted on the victim.

## Conclusions

Munchausen Syndrome by Proxy (MSPB) presents a complex diagnostic challenge that requires early recognition to prevent significant harm to the victim. MSPB is an uncommon diagnosis in dermatology; however, clinicians must be made aware of this diagnosis and the implications that can arise from MSBP if not diagnosed and treated promptly. It is essential to identify MSBP as the diagnosis and immediately involve a multidisciplinary team in the ethical and legal management of these cases to reduce the physical, psychological, and educational impact on the victim. While there are a variety of caregiver-patient relationships that can present with MSBP, it most often consists of a child and their guardian. Dermatologists, in particular, play a crucial role in recognizing cutaneous signs of MSBP, which are often key indicators of this condition. A multidisciplinary approach is necessary for proper diagnosis and management, ensuring the safety of the victim and addressing the psychosocial factors contributing to the caregiver's behavior. The involvement of healthcare providers, social services, and law enforcement is critical for intervening in these cases. Continued research and education are needed to develop standardized tools for diagnosis, prevention, and improving long-term outcomes for both victims and perpetrators.
